# A greater decline in female facial attractiveness during middle age reflects women’s loss of reproductive value

**DOI:** 10.3389/fpsyg.2014.00179

**Published:** 2014-02-28

**Authors:** Dario Maestripieri, Amanda C. E. Klimczuk, Daniel M. Traficonte, M. Claire Wilson

**Affiliations:** Institute for Mind and Biology, The University of ChicagoChicago, IL, USA

**Keywords:** facial attractiveness, middle age, sex differences, reproductive value, power, personality

## Abstract

Facial attractiveness represents an important component of an individual’s overall attractiveness as a potential mating partner. Perceptions of facial attractiveness are expected to vary with age-related changes in health, reproductive value, and power. In this study, we investigated perceptions of facial attractiveness, power, and personality in two groups of women of pre- and post-menopausal ages (35–50 years and 51–65 years, respectively) and two corresponding groups of men. We tested three hypotheses: (1) that perceived facial attractiveness would be lower for older than for younger men and women; (2) that the age-related reduction in facial attractiveness would be greater for women than for men; and (3) that for men, there would be a larger increase in perceived power at older ages. Eighty facial stimuli were rated by 60 (30 male, 30 female) middle-aged women and men using online surveys. Our three main hypotheses were supported by the data. Consistent with sex differences in mating strategies, the greater age-related decline in female facial attractiveness was driven by male respondents, while the greater age-related increase in male perceived power was driven by female respondents. In addition, we found evidence that some personality ratings were correlated with perceived attractiveness and power ratings. The results of this study are consistent with evolutionary theory and with previous research showing that faces can provide important information about characteristics that men and women value in a potential mating partner such as their health, reproductive value, and power or possession of resources.

## INTRODUCTION

Ratings of human facial attractiveness tend to be highly consistent across different cultures and socioeconomic backgrounds (e.g., Langlois et al., 2000; Little et al., 2011), suggesting that there is high agreement among individuals as to what constitutes a beautiful face. Since facial attractiveness represents an important component of an individual’s overall attractiveness as a potential mating partner, the study of facial attractiveness figures prominently in evolutionary research on mate attraction and mating strategies (e.g., Thornhill and Gangestad, 2008). Much of this research has focused on characteristics that influence judgments of facial attractiveness such as symmetry, averageness, femininity and masculinity, skin quality, and cues to personality or power (e.g., Thornhill and Gangestad, 1999; Rhodes, 2006; Little et al., 2011). Evolutionary studies have also addressed potential sources of individual differences in face preferences such as hormone levels and fertility, own attractiveness and personality, and previous experience or familiarity. Among the factors involved in judgments of facial attractiveness, age has been relatively neglected. For example, in recent reviews of the literature on evolutionary based research on facial attractiveness, the relationship between age and facial attractiveness is either not addressed at all or mentioned very briefly (e.g., Little et al., 2011).

Health gradually deteriorates with increasing age, therefore it is not surprising that younger and healthier people are preferred as potential mating partners, and therefore considered more attractive, by both men and women (see Kenrick and Keefe, 1992; Matts et al., 2007; Gray, 2012). Age is also a marker of fertility and reproductive value for women but not necessarily for men. When women reach menopause, at around 50 years of age, they cease to be viable mating partners regardless of their health, while men’s reproductive value can remain high for 2–3 more decades. Therefore, there may be little or no selective pressure to maintain high facial attractiveness in post-menopausal women, while facial attractiveness in older men could be a significant factor in their mating success (see Kenrick and Keefe, 1992). As a result, although both men and women should experience a gradual reduction in facial attractiveness through middle age, post-menopausal women should experience a greater reduction in facial attractiveness when compared to same-aged men (see also Alley, 1988, 2002).

Another factor could contribute to this hypothesized gender difference in age-related decline in facial attractiveness. In men, increasing age is generally associated with an increase in social status and resource possession, which in turn enhances their value and attractiveness as potential mating partners, while this occurs to a much lesser degree in women. Thus, in men, a health-related decline in facial attractiveness through middle age should be partially compensated by a status- and resource-related increase in facial attractiveness, while no such compensation is expected in women. This, in addition to changes in female reproductive value, should contribute to the greater decline in facial attractiveness in postmenopausal women when compared to same-aged men.

Although the above hypotheses have been largely overlooked by recent evolutionary based research on facial attractiveness, a number of earlier empirical studies investigated age-related changes in ratings of facial attractiveness in men and women (Cross and Cross, 1971; Berman et al., 1981; Korthase and Trenholme, 1982; Wernick and Manaster, 1984; Mathes et al., 1985; Deutsch et al., 1986; Henss, 1991; McLellan and McKelvie, 1993; Wilcox, 1997; Perlini et al., 1999; Teuscher and Teuscher, 2007). These studies, however, did not include quantitative comparisons between the two genders or an explicit test of the hypothesis that post-menopausal women should experience a greater reduction of facial attractiveness relative to same-aged men. In addition, with one or two exceptions, the authors of these studies did not discuss their findings from an evolutionary perspective but mainly speculated on socially constructed standards of beauty and environmental influences. Therefore, the question of whether changes in facial attractiveness in middle-aged women and men reflect changes in their reproductive value, perceived power, and overall attractiveness as potential mating partners remains unanswered.

In this study, we investigated ratings of facial attractiveness and power in two groups of middle-aged women (35–50 years and 51–65 years), which roughly correspond to pre- and post-menopause (National Institute on Aging, 2008), and two groups of same-aged men. Faces were rated by middle-aged women and men (see Methods). We tested three main hypotheses: (1) that perceived facial attractiveness would be lower for older than for younger men and women; (2) that the age-related reduction in facial attractiveness would be greater for women than for men; and (3) that for men, there would be a larger increase in perceived power at older ages. For hypothesis 1, we anticipated that male and female raters would contribute similarly. However, we anticipated that male raters would make a greater contribution to the effect expected in hypothesis 2, while female raters should make a greater contribution to the effect expected in hypothesis 3. Finally, since ratings of facial attractiveness and power may be influenced by perception of age and personality, we asked the raters of the face stimuli to estimate the age and the personality characteristics of the stimuli.

## MATERIALS AND METHODS

### FACE STIMULI

Eighty photographs of individuals’ faces (52 males, 28 females) were obtained from academic web pages at the authors’ own institution. Most of these photos were taken by a university photographer, therefore the images were relatively standardized. Only high-quality front-facing photos were selected. The photos were edited with Adobe Acrobat to show only the head, eliminating any clothing, and background. Age of the face stimuli was obtained from information publicly available online. Age of face stimuli ranged from 35 to 65 years (mean + SE = 51.42 + 0.87 years; males = 51.28 + 1.07; females = 51.67 + 1.54). Since the authors had met in person all the individuals in the photographs at least once in the 6-month period prior to this study, it was possible to verify that the photographs had been taken recently. Photographs that portrayed individuals at a much younger age were not included in the stimulus set. Sixty-seven faces were Caucasian and 13 were of other ethnicities. All face stimuli were rated by two independent observers and there was 100% agreement between them as to whether or not the face stimulus was smiling. The distribution of smiling and non-smiling face stimuli did not differ significantly in relation to the age and sex stimulus categories.

### ONLINE SURVEYS

The face stimuli were individually presented in an online survey using the web-based survey provider Qualtrics (). The order of stimulus presentation was randomized for each respondent. Respondents were asked to estimate the age of the face stimulus, rate facial attractiveness from 1 to 10 (1 being very unattractive and 10 very attractive), rate how powerful the person was (how much influence the person has over other people) on a 1 to 5 scale (1 least powerful, 5 most powerful), and rate their personalities. For each of the Big Five personality dimensions, respondents were given dichotomous forced choices to the question “Which of the following personality traits do you think would best describe this person?” The options were as follows: for Openness to Experience, inventive/curious or consistent/cautious; for Conscientiousness, efficient/organized or easy-going/careless; for Extraversion, outgoing/energetic or solitary/reserved; for Agreeableness, friendly/compassionate or cold/unkind; and for Neuroticism, sensitive/nervous or secure/confident. There was no time constraint on completing the survey but respondents were required to rate all stimuli and answer all questions.

### RESPONDENTS

Respondents were 60 (30 males, 30 females) individuals ranging in age between 25 and 55 years (mean + SEM = 39.60 + 0.90; males = 39.93 + 1.31; females = 39.26 + 1.26). Respondents were middle-aged but younger than the face stimuli in an effort to select for potentially reproductively active individuals (e.g., there were no post-menopausal women among the raters) who could view the face stimuli as potential mating partners. Fourteen respondents (seven males, seven females; age range: 25–55) were initially recruited for a pilot study by word of mouth. An additional 46 respondents (23 males, 23 females; age range 30–50) were obtained through Qualtrics. All respondents were heterosexual and, with two exceptions, Caucasian. All were blind to the study’s main hypotheses. Respondents completed an informed consent form before participating in the study. The study was approved by the IRB.

## RESULTS

There was a highly significant positive correlation between the face stimuli’s estimated ages and their real ages (*N* = 80; *r* = 0.72; *p* < 0.0001), suggesting that the respondents were generally accurate in estimating the stimuli’s ages. All data analyses provided similar results whether data on real age or estimated age were used, therefore to avoid redundancy, only data analyses involving real age data are presented. For analysis purposes, the face stimuli were divided into two age groups, younger (35–50 years; *N* = 37; 25 males, 12 females) and older (51–65 years; *N* = 43; 27 males, 16 females). There was no significant difference in the relative proportion of male and female stimuli in the two age groups (χ^2^ = 0.19; *df* = 1; NS) and no significant gender difference in age in each group (younger: males = 44.64 ±0.92; females = 44.16 ± 1.68; *t*-test for unpaired samples; *t* = 0.27; *df* = 35; NS; older: males = 57.44 ± 0.78; females = 57.31 ± 1.01; *t* = 0.10; *df* = 41; NS).

**FIGURE 1:**
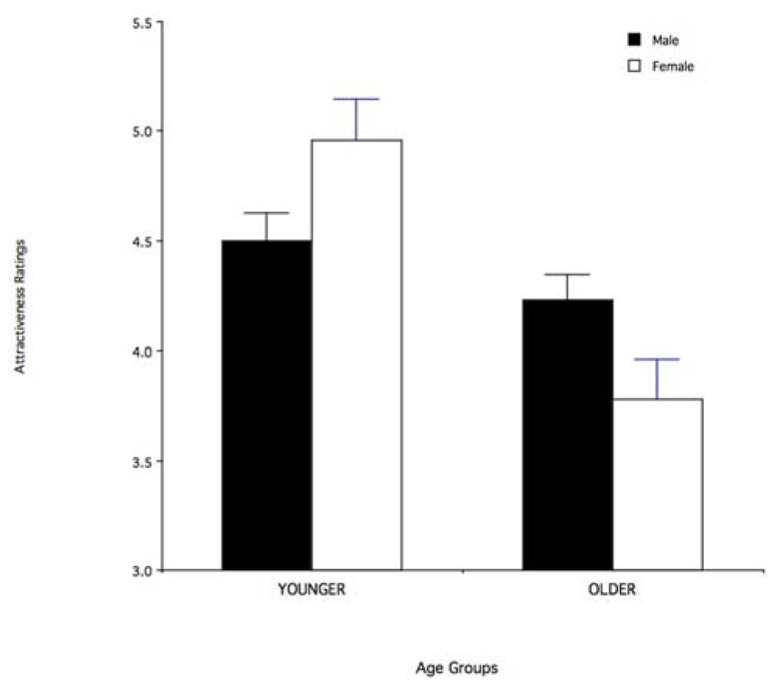
**Mean (+SEM) ratings of facial attractiveness for male and female face stimuli in the younger (35–50 years) and older (51–65 years) age groups.** Data for male respondents only.

A 2 × 2 × 2 ANOVA was used to analyze the effects of stimulus sex (male, female), stimulus age (young, old), and respondent sex (male, female). There was a significant main effect of stimulus age, with older stimuli being generally rated as less attractive than younger stimuli [*F*(1,76) = 12.28; *p* = 0.0008]. There was also a significant three-way interaction between stimulus age, stimulus sex, and respondent’s sex [*F*(1,76) = 4.05; *p* < 0.05). *Post hoc* tests with the Bonferroni correction indicated that, for male respondents, reduction in attractiveness with older ages was larger for the female than for the male stimuli (*p* < 0.05; see **Figure 1**), and that the effect of stimulus age on male respondents’ ratings of female stimuli was greater than the effect of stimulus age on female respondents’ ratings of male or female stimuli (*p* < 0.05). In other words, the age-related reduced attractiveness was greater for female than for male stimuli and this effect was mainly driven by male respondents.

**FIGURE 2:**
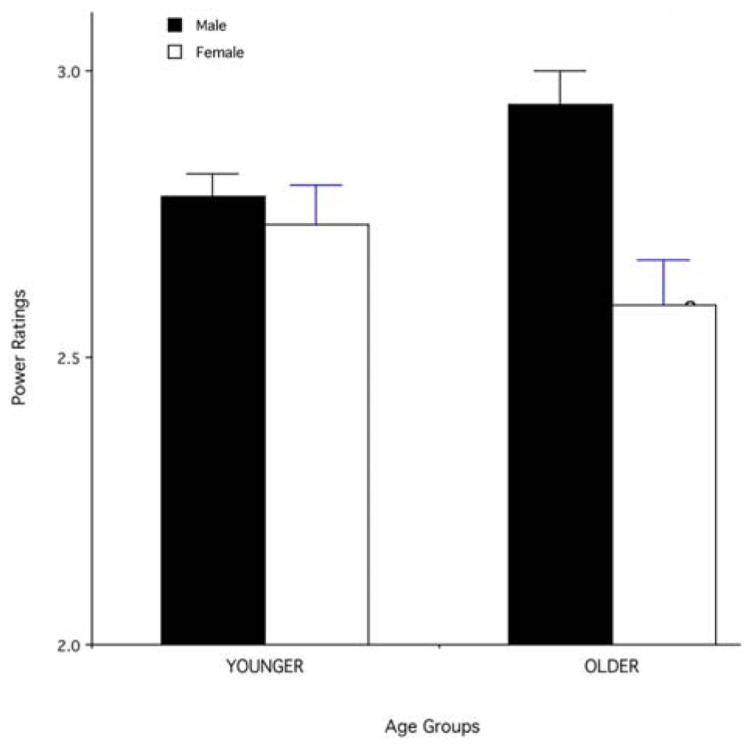
**Mean (+SEM) ratings of power for male and female face stimuli in the younger (35–50 years) and older (51–65 years) age groups.** Data for female respondents only.

A similar 2 × 2 × 2 ANOVA was also used to analyze the ratings of perceived power. The analysis revealed a significant main effect of gender [*F*(1,76) = 11.82, *p* = 0.001], with males being rated as more powerful than females, and a significant three-way interaction between stimulus age, stimulus sex, and respondent’s sex [*F*(1,76) = 3.97, *p* = 0.05). *Post hoc* tests with the Bonferroni correction indicated that the effect of stimulus age on ratings of perceived power of male and female stimuli was greater for female than for male respondents (*p* < 0.05); specifically, for female respondents, older male stimuli were rated as more powerful than younger male stimuli while the opposite was true for female stimuli (**Figure 2**). For male respondents, the effect of stimulus age on the rating of perceived power of male and female stimuli was in the same direction as in the female respondents, but much weaker and non-significant (*p* > 0.05). In other words, there was an age-related increased perceived power for male but not for female stimuli and this effect was mainly driven by female respondents.

Power ratings were significantly positively correlated with ratings of attractiveness for both male (*N* = 52; *r* = 0.45; *p* = 0.0008) and female stimuli (*N* = 28; *r* = 0.67; *p* < 0.0001). This was also true for male and female respondents considered separately. Power ratings, however, were not significantly correlated with age in either male (*N* = 52; *r* = 0.16; NS) or female stimuli (*N* = 28; *r* = 0.13; NS), or across all stimuli (*N* = 80; *r* = 0.04; NS). Similar non-significant results were obtained if data for male and female respondents were analyzed separately.

**FIGURE 3:**
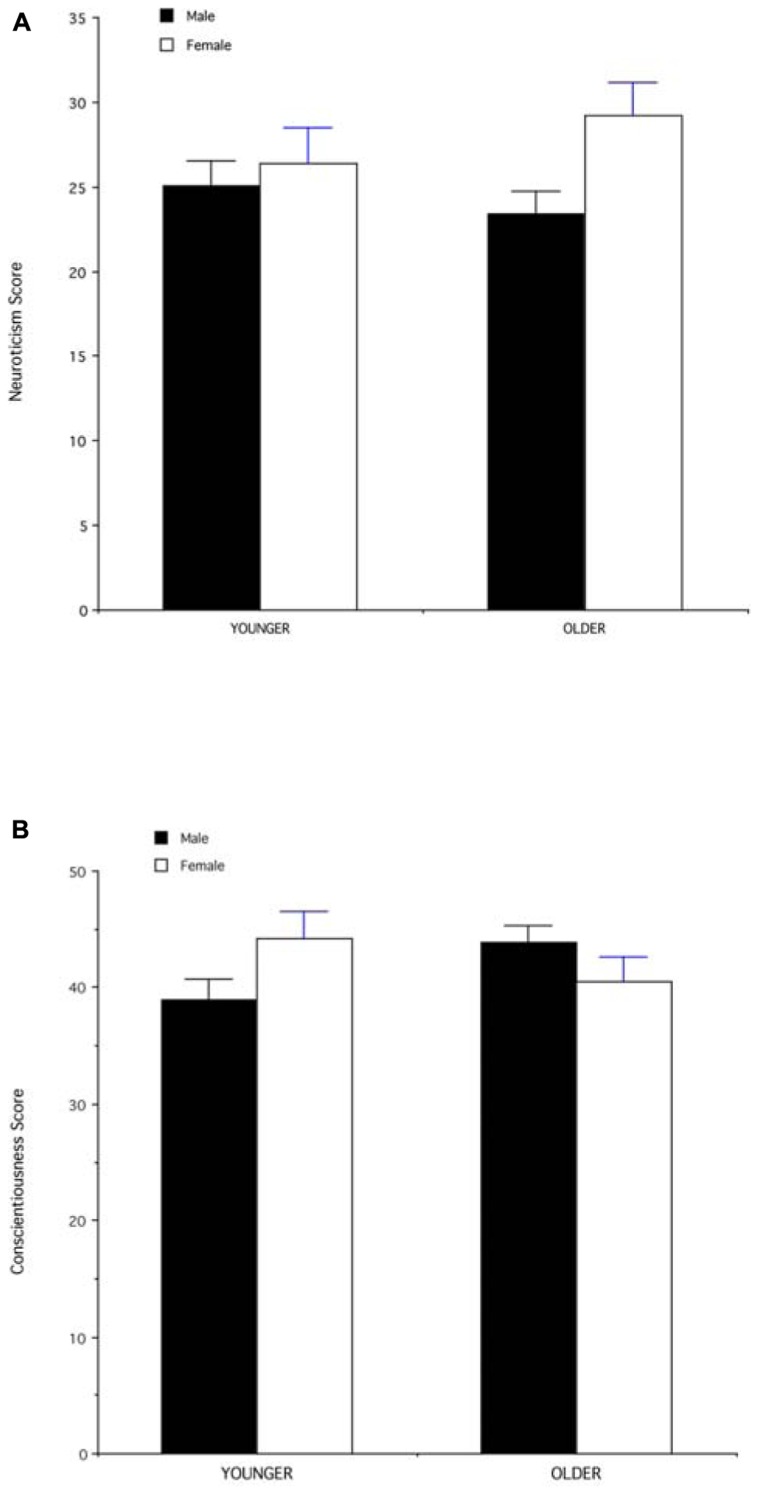
**(A)** Mean (+SEM) values of neuroticism score (number of respondents who rated stimuli as secure/confident) for male and female face stimuli in the younger (35–50 years) and older (51–65 years) age groups. **(B)** Mean (+SEM) values of conscientiousness score (number of respondents who rated stimuli as efficient/organized) for male and female face stimuli in the younger (35–50 years) and older (51–65 years) age groups.

The ratings of the five personality dimensions by male and female respondents were highly positively correlated (all *p* values < 0.0001), although male respondents generally rated stimuli as being more conscientious and more outgoing than females did (conscientiousness: males = 21.45 + 0.48; females = 20.21 + 0.58; paired *t*-test = 3.17; *df* = 79; *p* = 0.002; extraversion: males = 15.77 + 0.62; females = 14.07 + 0.79; *t*-test = 4.25; *p* < 0.0001). Two personality dimensions were associated with perceived power: conscientiousness and neuroticism. Power was positively correlated with conscientiousness (*N* = 80; *r* = 0.37; *p* = 0.0006), such that more conscientious people were rated as more powerful, while more easy-going people were rated as less powerful. Power was negatively correlated with neuroticism (*N* = 80; *r* = -0.67; *p* < 0.0001) such that more secure people were rated as more powerful, while more neurotic people were rated as less powerful. Neuroticism showed a significant main effect of gender [*F*(1,76) = 4.27; *p* < 0.05), with males rated as more secure than females, but no significant interaction between gender and age [*F*(1,76) = 1.6; NS; **Figure 3A**). Conscientiousness showed no significant main effect of gender [*F*(1,76) = 0.1; NS], but a significant interaction between gender and age [*F* = 4.65; *p* < 0.05; **Figure 3B**), such that older males were rated as more conscientious, while older females were perceived as more easy-going.

Higher facial attractiveness was associated with four different personality traits: openness to experience (*N* = 80; *r* = 0.39; *p* = 0.0003); extraversion (*N* = 80; *r* = 0.56; *p* < 0.0001); friendliness (*N* = 80; *r* = 0.42; *p* < 0.0001); and self-confidence or security (*N* = 80; *r* = 0.54; *p* < 0.0001). With the exception of neuroticism, which showed a main effect of gender, none of these personality characteristics associated with attractiveness differed significantly in relation to age or gender.

Finally, ratings of three personality dimensions (openness to experience, extraversion, and agreeableness) differed in relation to whether or not the face stimulus was smiling (smile: *N* = 65; no smile: *N* = 15). Face stimuli with smiles were rated as more inventive (*t* = 4.56; *df* = 78; *p* < 0.0001), more extraverted (*t* = 6.35; *df* = 78; *p* < 0.0001), and friendlier (*t* = 8.98; *df* = 78; *p* < 0.0001) than face stimuli without smiles. Therefore, smiling faces were generally attributed more positive personality traits and higher attractiveness. In contrast, for the two personality dimensions (conscientiousness and neuroticism) that were associated with perceived power and were likely to account for the observed age and gender differences in facial attractiveness, there were no differences in relation to whether or not the face stimuli were smiling.

## DISCUSSION

In this study, the respondents were generally accurate in estimating the stimuli’s ages from the photos of their faces and there was a strong negative association between age and perceived attractiveness for both male and female face stimuli, suggesting that any age-related decrease in skin quality or health occurring in the 35–65 year age period negatively impacts both male and female facial attractiveness (see also Fink et al., 2006; Matts et al., 2007; Gray, 2012). A negative association between age and facial attractiveness has been reported by previous studies (e.g., Korthase and Trenholme, 1982; Wernick and Manaster, 1984; Mathes et al., 1985; Henss, 1991; McLellan and McKelvie, 1993; Teuscher and Teuscher, 2007), although the methodology used in these studies was quite heterogeneous and not directly comparable to that of the present study.

Consistent with our hypothesis, the decline in facial attractiveness of 51–65 year old (and presumably post-menopausal) women relative to 35–50 year old (and presumably pre-menopausal) women was significantly larger than the decline in facial attractiveness for the corresponding male age groups. Although this effect was significant across both male and female respondents, it was mainly driven by male respondents (suggestive but inconclusive evidence for a similar effect was also provided by Mathes et al., 1985; Deutsch et al., 1986; Henss, 1991; McLellan and McKelvie, 1993; but see Zebrowitz et al., 1993; Teuscher and Teuscher, 2007; for negative evidence). This result suggests that men are particularly attuned to age-related changes in female facial attractiveness during middle age. Although it is unclear whether or not there is selective pressure to maintain high facial attractiveness in post-menopausal women, there should be pressure on men to visually discriminate between pre-menopausal and post-menopausal middle-aged women (see also Wildt and Sir-Petermann, 1999) because these women clearly differ in their reproductive value and therefore in their viability as potential mating partners.

We hypothesized that perceived power would be positively associated with facial attractiveness and that for men, an increase in perceived power with older ages would partially compensate for their age-related decline in facial attractiveness. These hypotheses were supported by our data. Male and female face stimuli that were rated higher in perceived power were also rated higher in attractiveness, regardless of the stimuli’s age and the rater’s gender. Perceived power in itself was not significantly correlated with age, in part because the relation between power ratings and age was different for male and female stimuli. While perceived power ratings were higher for 51–65 year old men than for their younger counterparts, older women were rated as less powerful than younger women. The effect of increased perceived power for older men was mainly driven by female respondents. It may be argued that since men’s social status and possession of resources play a central role in female mating strategies (e.g., Kenrick and Keefe, 1992), women should be particularly attuned to men’s facial cues that are indicative of power and able to recognize these cues in older middle-aged men (see also Keating, 1985; Cunningham et al., 1990; Boothroyd et al., 2007; Neave and Shields, 2008).

Male face stimuli, in general, received higher ratings of perceived power than female stimuli, regardless of the stimuli’s age and the rater’s gender. Perceived power ratings were correlated with some personality ratings. Specifically, neuroticism was negatively correlated with perceived power, and male stimuli were perceived to be more secure and self-confident than female stimuli, regardless of age. Perceived power was also associated with conscientiousness. Face stimuli that were perceived to be more conscientious also received higher ratings of perceived power. Conscientiousness was not different in male and female stimuli, but there was a significant interaction between gender and age, such that older males were rated as more conscientious, while older females were rated as less conscientious. This effect may explain why perceived power ratings increased with age for males but decreased for females.

Although we offered no direct evidence that perceived personality ratings mediated the relationship between perceived power and perceived attractiveness, correlational analyses indicated that perceived conscientiousness and neuroticism were correlated with perceived power (but not with attractiveness), and that perceived power in turn was correlated with perceived attractiveness. Other personality ratings were correlated with ratings of attractiveness such that face stimuli that were rated higher in openness to experience, extraversion, friendliness, and self-confidence were also rated higher in attractiveness. Aside from the previously mentioned gender difference in perceived neuroticism, none of these personality dimensions showed significant gender differences or significant age × gender interactions. Therefore, although “positive” personality traits (inventive, extraverted, friendly, and secure) are associated with higher attractiveness ratings, these measures were unlikely to account for the observed age- and gender-related differences in attractiveness.

This study is one of the few evolutionary investigations of facial attractiveness in middle age and the first one providing clear evidence that post-menopausal women experience a greater age-related decline in facial attractiveness than same-aged men. The findings of this study are generally consistent with the notion that facial attractiveness in middle age reflects different aspects of “quality,” some of which are similar for men and women (e.g., age-related changes in health) and others that are different for the two genders (age-related changes in reproductive value for women and in power or status in men; see Hume and Montgomerie, 2001). Furthermore, we provided evidence that some perceived personality traits are correlated with perceived attractiveness and power ratings in young and old male and female stimuli. Future research should better incorporate and integrate, both conceptually and empirically, the study of facial attractiveness in relation to age, especially in middle age, into the investigation of mate attraction and mating strategies in men and women. Future research should also further investigate the mechanisms responsible for sex differences in the perception of attractiveness and power in male and female face stimuli in relation to their age.

## Conflict of Interest Statement

The authors declare that the research was conducted in the absence of any commercial or financial relationships that could be construed as a potential conflict of interest.
